# Optimizing PMTCT efforts by repeat HIV testing during antenatal and perinatal care in resource-limited settings: A longitudinal assessment of HIV seroconversion

**DOI:** 10.1371/journal.pone.0233396

**Published:** 2020-05-29

**Authors:** Ivy Mushamiri, Maureen Adudans, Donald Apat, Yanis Ben Amor

**Affiliations:** 1 Department of Epidemiology, Mailman School of Public Health, Columbia University, New York, NY, United States of America; 2 UNICEF, Child Health and Community Platforms, Nairobi, Kenya; 3 Columbia Global Centers East and Southern Africa, Nairobi, Kenya; 4 Center for Sustainable Development, Earth Institute, Columbia University, New York, NY, United States of America; University of Namibia, NIGERIA

## Abstract

**Background:**

Mother to child transmission (MTCT) of HIV remains a challenge in resource-limited settings. Central to elimination of MTCT is effective Provider Initiated HIV Counseling and Testing (PICT). Research has shown that conducting PICT only at the initial antenatal care (ANC) visit fails to benefit pregnant women who seroconvert later in their pregnancy. This study aimed to determine the most cost effective time to perform repeat HIV testing during ANC and perinatal care (PNC).

**Methods:**

We studied the repeat HIV testing results of pregnant women ≥ 18 and adolescent girls aged 15–17 in the Sauri, Kenya Millennium Villages Project (MVP) site. Nurses provided HIV screening to 1,403 expectant women and 256 adolescent girls following the 1^st^, 2^nd^, 3^rd^ and 4^th^ ANC visits, at birth and 6 and 14 weeks postpartum.

**Results:**

Five women seroconverted during the study period (incidence proportion 0.41%). One woman seroconverted at the 2^nd^ ANC visit, another one at the 3^rd^, two at the 4^th^ and one at 6 weeks post-partum. Of all the women who seroconverted, four reported an HIV negative primary partner, while one reported an unknown partner status. None of the participants reported condom use during pregnancy. Two of the seroconverters vertically transmitted HIV to their babies. The results did not suggest a clear pattern of seroconversion during ANC and PNC.

**Conclusions:**

The low rates of seroconversion suggest that testing pregnant women multiple times during ANC and PNC may not be cost effective, but a follow-up test during birth may be protective of the newborn.

## Background

Global scale-up of interventions for the prevention of mother-to-child HIV transmission (PMTCT) has led to a remarkable 70% reduction in new HIV infections among children worldwide since the year 2000 [1]. Much of this success is the result of enhanced HIV case detection and antiretroviral therapy (ART) coverage among pregnant women receiving antenatal care (ANC) in sub-Saharan Africa [1]. However, further effort is needed in order to reach the United Nations Sustainable Development Goals target of ending the AIDS epidemic by 2030 [2].

Undetected new, acute HIV infection during pregnancy and the postpartum period remains a significant challenge to PMTCT efforts in sub-Saharan Africa [3–5]. Acute HIV infection is associated with increased risk of vertical HIV transmission during pregnancy, delivery and breast-feeding than chronic infection because of increased viral loads [6–8]. A mathematical model showed that the proportion of MTCT from pregnant women who seroconverted after their first ANC visit was 26% (95% CI 22–30%) in 2008 [9], but this number could be reduced by targeted interventions. HIV testing during ANC in this region continues to rely on rapid diagnostic tests (RDTs) for HIV serology and may miss women presenting to ANC during the first two weeks post-exposure where antibodies made against the HIV infection are not detected by RDTs [10]. Furthermore, women in the region are at an increased risk for new HIV infection during pregnancy and the postpartum period [11]. A meta-analysis done on studies of incident HIV infection during pregnancy and the post-partum period showed that the pooled incident rate during pregnancy in sub-Saharan Africa was 4.7 per 100 person-years (95% CI 3.3–6.1), showing a disproportionate risk in the region compared to the global risk of 3.8 per 100 person-years (95% CI 3.0–4.6) both during pregnancy and the post-partum period [11]. This increase in risk can be explained by both behavioral and biological factors such as the changes in sexual practices of partners during pregnancy and the post-partum period [11–13] and changes in the genital mucosal surfaces or HIV target cells due to hormonal changes during pregnancy which make pregnant women more susceptible to HIV acquisition [14–16]. Current guidelines target such incident cases by recommending repeat HIV testing during pregnancy and/or the postpartum period, as well as early partner testing[17–19]. In 2012, Kenya adopted the international guidelines that stipulated that HIV negative pregnant women should be retested for HIV after three months [18], but shortages in rapid HIV test kits and other resources make adherence to such guidelines difficult. A study done among 2,145 pregnant women attending an ANC clinic at a large district hospital in southwest Kenya identified key barriers to retesting such as an inability to return to the clinic and late gestation age (>28 weeks) at first ANC visit [20].

There is a growing body of research aimed at better understanding the risk factors for new, acute HIV infection during pregnancy and the postpartum period [3–5, 11, 20–22], yet risk-based guidelines for prioritized utilization of rapid HIV test kits in a limited resource setting such as Kenya do not exist [17–19]. When test kits are scarce, it is often difficult for clinicians in the field to prioritize partner testing versus repeat testing for pregnant women, to decide the best timing of repeat testing, or to extend testing to other groups, such as adolescents. We therefore conducted a prospective cohort study investigating the incidence and timing of HIV seroconversion during pregnancy and the postpartum period, along with associated risk factors, in order to help inform such guidelines in the future.

### Study setting

#### Millennium villages project

**Fig 1:**
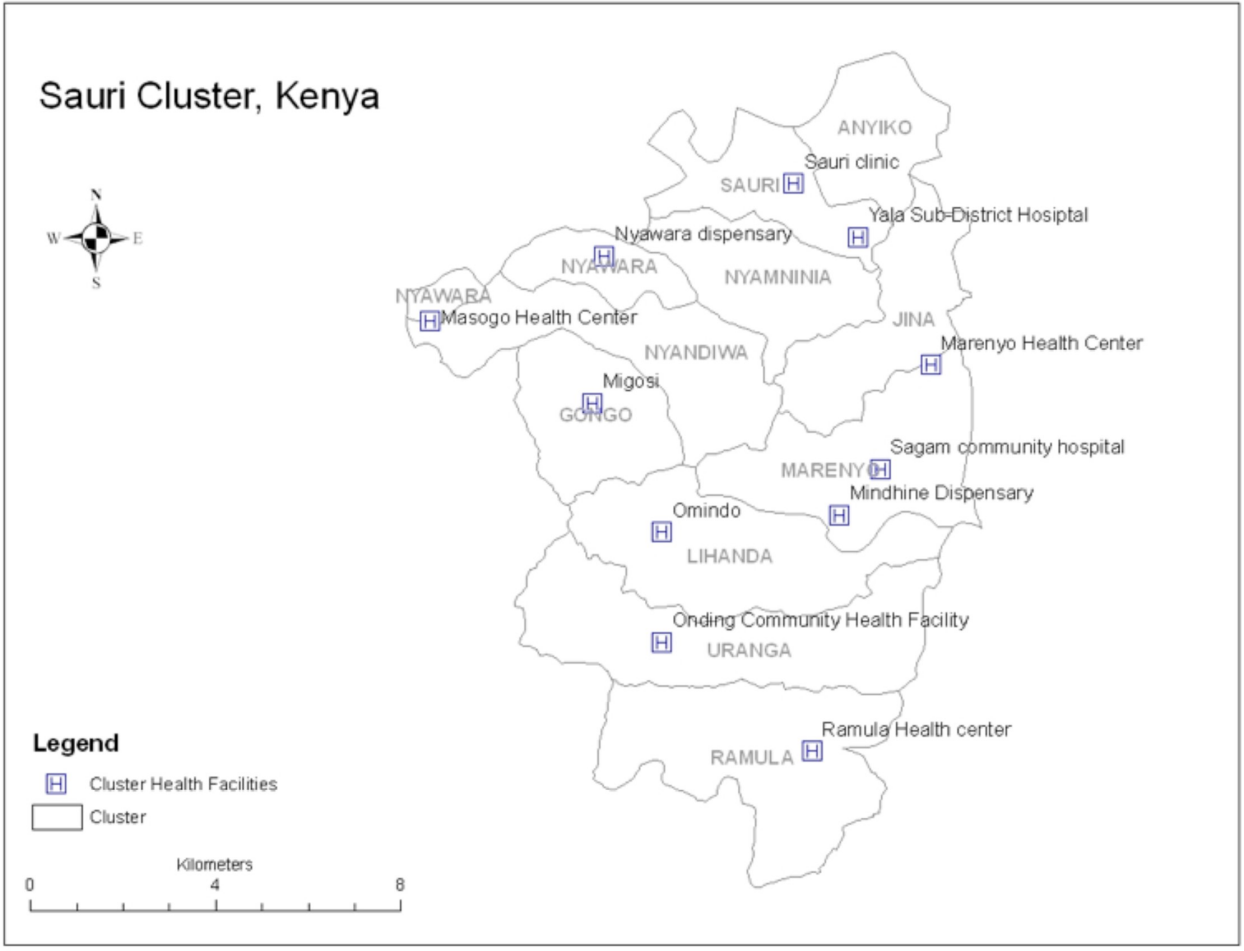
Map of the Sauri cluster. The MVP site in Kenya is divided into 12 sub-locations including Sauri, and is dubbed “Sauri Cluster” because Sauri was where the inception of the package of MVP comprehensive development interventions was first implemented. Each sub-location with its own network of CHWs and the Cluster has 10 health facilities in total, including a sub-district referral hospital.

This longitudinal study recruited pregnant women and adolescents who were residents of Millennium Villages Project (MVP) in Sauri cluster (see Fig 1) in Siaya County, Western Kenya. The Millennium Villages Project [23] was a rural development project designed as a proof of concept on how to achieve the Millennium Development Goals (MDGs) with the ultimate aim of eradicating extreme poverty. MVP’s novelty was in its ‘integrated approach’ to development, whereby science and community-based investments are made simultaneously into key sectors: Agriculture and Business Development, Education, Infrastructure, Health, Environment and Gender. By so doing, the project and its beneficiaries are able to maximize and build upon the strong synergies that exist across these key sectors. Within each of the sectors, a large number of services are delivered through a strong collaboration between community, government and partners. In 2009, MVP and UNAIDS entered into a collaborative agreement to reach “MTCT-free” zones in the MVP sites, including Sauri, Kenya. The MTCT-free zones project was based on UNAIDS’ “Global Plan towards the elimination of new HIV infections among children by 2015 and keeping their mothers alive” [24]. Scientific-based approaches aligned to national and WHO policies were designed for each of the four pillars of PMTCT [25]. These approaches were adapted to suit the host community culture and needs specific to each of the sites, and were prioritized for implementation alongside novel practices. It was envisioned that the UNAIDS-MVP partnership would harness the benefits of an integrated and comprehensive rural development project, which encompasses the strengthening of the primary health care system and would plug these into the implementation of the four pillars of PMTCT.

#### Siaya District and Kenya national guidelines

HIV prevalence in Siaya is 4.2 times higher than the national prevalence at 24.8% (Kenya HIV estimates, 2015), with an estimated 796 new HIV infections annually in 2015 among children aged 0–14 years [26]. Testing in ANC is offered on an “opt-out” basis and uptake in Sauri is very high with <1% declining testing in 2009. All 10 clinics in the Sauri site (Fig 1) provide rapid HIV testing, and Yala District Hospital, the referral site, offers CD4 monitoring. With the introduction of Option B+ in 2014, PMTCT coverage still remained at sub-optimal level with coverage of 57% in Siaya county in 2015 (compared to the national estimate of PMTCT coverage of 75%), contributing to 20.6% of exposed children infected with HIV in 2015 [26]. All 10 facilities within the Sauri cluster adopted Option B+ in mid-2014 as part of the PMTCT services program following recommendations from the Ministry of Health (MOH). Survey data from 2017 suggest approximately 75% of women are tested for HIV during their pregnancy, compared to 65% at the time of our study in 2014 (internal MVP data).

## Methods

### Study design

Women who presented to the 10 health facilities in Sauri between January 2013 and November 2015 for ANC were offered HIV testing using RDTs following the national algorithm as part of the routine care. Each mother-baby pair was followed for 18 months post-delivery, until the time when the infant had the final antibody test to determine whether there was vertical transmission. Those who tested HIV negative and consented to participate in the study were verbally administered a questionnaire and demographic and clinical information was entered into a longitudinal register. More information was collected in subsequent ANC and PNC visits up to 14 months post-partum. Participants were tested for HIV at each ANC and PNC visit and at delivery.

### Study population

Trained nurses screened all pregnant women presenting to their first ANC visit for eligibility. Eligibility criteria included: negative HIV status; age 15 or above; resident status within the Sauri cluster. Nurses informed eligible pregnant women of the study’s aims, procedures, risks and benefits. Interested adult pregnant women (age 18 and above) were enrolled in the study after signing a standardized consent form. Participants less than 18 years of age who are parents, guardians, married or co-habiting are, according to the national HTC guidelines [27], termed “mature minors” and were taken through the informed consent process independently. Participants aged 15–17 who were not mature minors but interested in the study were asked to return with a parent/guardian to obtain parental/guardian consent after which assent was sought from the adolescent. Based on the HIV incidence rate of Kenya, we set an enrollment goal of 2,100 participants. Study retention efforts included conducting home visits for women who missed their ANC or PNC appointments, via community health workers (CHWs) linked to the health facilities according to their catchment area, to encourage them to present to the facility for ANC/PNC and conducting a final home or facility-based HIV test via trained HIV counselors. In the Sauri cluster, 158 CHWs covered 14,500 households at a ratio of 1 CHW to 120 households [28, 29].

All women who tested positive for HIV during the course of our study were enrolled into HIV care.

### Outcomes

The primary outcome of interest was HIV seroconversion at subsequent ANC and PNC visits. Other outcomes assessed were timing of ANC visits, sexual activity during pregnancy and postpartum and post-partum family planning use.

### Additional covariates

Other covariates assessed were gestation period at first ANC visit, age, marital status, reported partner’s HIV status, and syphilis history. For a sub-sample of participants (all participants who seroconverted during the study and a small proportion of those who completed the study), additional covariates were also collected such as length of marriage, condom use during pregnancy, malaria infection during pregnancy, updated reported partner’s HIV status, and ART status for eligible HIV positive participants.

### Statistical analysis

All statistical analyses were conducted using SAS 9.4 software and Microsoft Excel 2011. We anticipated enrolling 1,100 women and 1,000 adolescents (2,100 total) in order to have sufficient power to conduct a chi-square test to determine whether the distribution of new HIV cases varied by a statistically significant amount across time points [30] and be able to detect at least 5 cases at each time point should the incidence be low. HIV incidence proportion was determined by dividing the number of cases over the total number of participants with a final HIV status outcome and multiplying by 100 to get a percentage.

### Ethics approval and consent to participate

The study protocol was approved by the institutional review board of Columbia University in the City of New York. Columbia IRB approval AAK3403 and AAK2659. In Kenya, the study was approved by the Kenyatta National Hospital/ University of Nairobi-Ethics Review Committee KNH-ERC/A/2 and the protocol number is P477/08/2012. Participants were consented prior to enrollment using consent forms approved by Columbia University and Kenyatta National Hospital Ethical Review Boards. Interested adult pregnant women (age 18 and above) were enrolled in the study after signing a standardized consent form. Participants less than 18 years of age who are parents, guardians, married or co-habiting are, according to the national HTC guidelines [27], termed “mature minors and were taken through the informed consent process independently. Participants aged 15–17 who were not mature minors but interested in the study were asked to return with a parent/guardian to obtain parental/guardian consent after which assent was sought from the adolescent.

## Results

### Study demographics

The study enrolled 1,403 participants (256 adolescent girls and 1,143 adult women—4 unknown age). Demographic results are presented in Table 1. Median age was 21 (IQR 18–26). For the 256 adolescents enrolled, median age was 16 (IQR 16–17). The number and proportion of women married was 1,034 (73.70%). Only 9 (0.64%) participants reported an HIV positive partner and 491 (35.00%) did not know their partner’s HIV status at any point throughout the study period. The number of participants who had family planning administered at 6 weeks post-partum was 989 (70.49%). The majority of women (1066 [70.49%]) attended their first ANC visit during the second semester of their pregnancy. About half the participants presented for four ANC visits. Only 485 (34.57%) of women reported sexual activity during pregnancy and 994 (70.85%) between birth and 6 weeks post-partum.

**Table 1:** Demographic and clinical characteristics of participants (N = 1,403).

Characteristic		Median (IQR) or Count	Percent
Age (years)		21 (18–26)	
Missing		4	
Marital Status			
	Married	1034	73.70
	Polygamous Married	24	1.71
	Cohabiting	2	0.14
	Widowed Cohabiting	1	0.07
	Divorced Cohabiting	1	0.07
	Widowed	4	0.29
	Single	307	21.88
	Missing	30	2.14
Reported Partner HIV Status			
	Positive	9	0.64
	Negative	903	64.36
	Missing	491	35.00
Syphilis History	Yes	66	4.70
	No	1323	94.30
	Missing	14	1.00
Family Planning Administration at 6Wks PNC			
	Yes	989	70.49
	No	141	10.05
	Missing	273	19.46
Trimester of Pregnancy at 1st ANC Visit			
	First	143	10.19
	Second	1066	75.98
	Third	192	13.68
	Missing	2	0.14
Number of ANC Visits			
	4	738	52.60
	Less than 4	665	47.40
Sexual Activity During Pregnancy			
	Active	485	34.57
	Inactive	710	50.60
	Missing	208	14.83
Sexual Activity Between Birth and 6Wks PNC			
	Active	994	70.84
	Inactive	228	16.25
	Missing	181	12.90
Participant HIV status at 14 Weeks PNC			
	Positive	5	0.36
	Negative	1223	87.17
	Missing	175	12.47

### Study seroconversions

Final HIV status was ascertained for 1,228 (87.53%) participants, of which 213 (17.3%) were adolescents. Due to the very small number of participants testing positive for the primary outcome, multivariable analysis was not conducted and only descriptive statistics were calculated.

**Fig 2:**
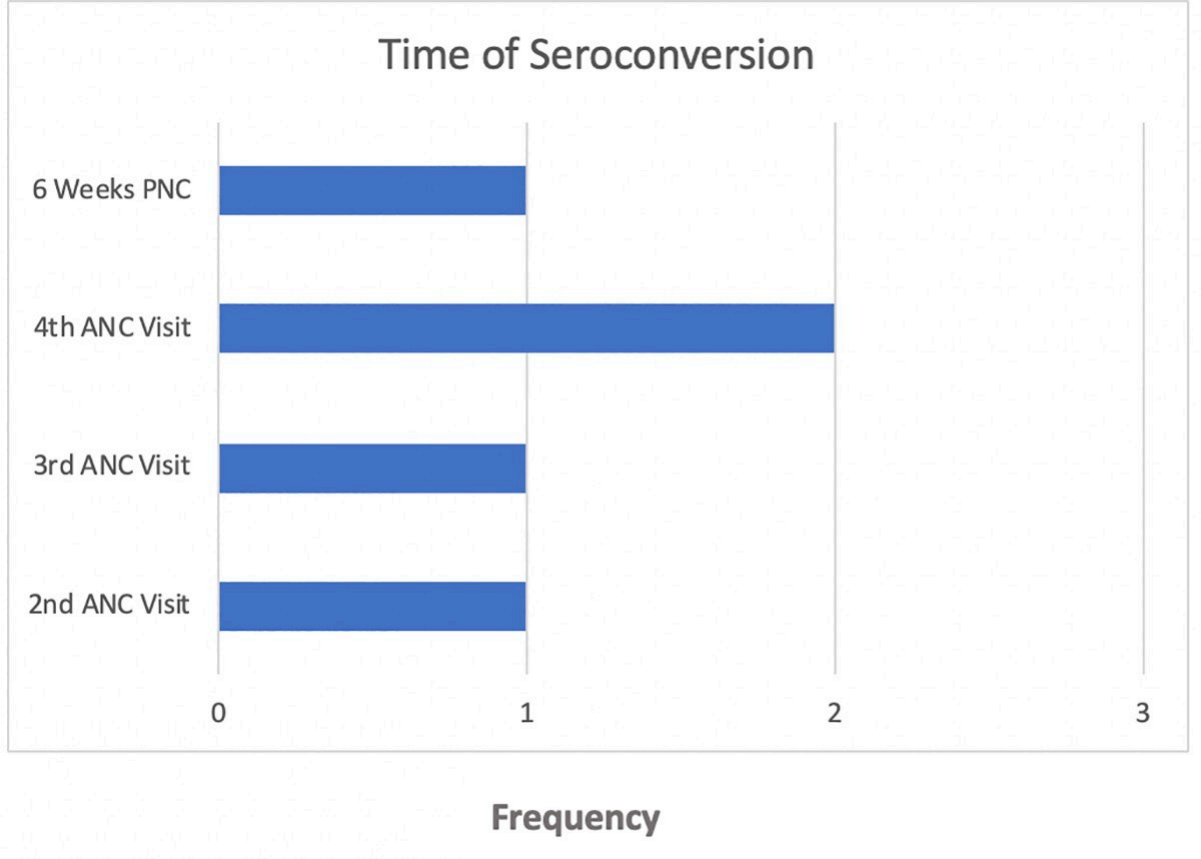
Time of seroconversion among participants.

During our study, one woman tested HIV positive at the 2^nd^ ANC visit, one at the 3^rd^, two at the 4^th^ and one at 6 weeks post-partum (see Fig 2). There were 5 total serocoversions (out of the 1,228 with a known final HIV status at 14 weeks post-partum), giving an incidence proportion of 0.41%.

Of all the women who seroconverted during our study, four reported an HIV negative primary partner, while one reported an unknown partner status. None of the participants reported condom use during pregnancy. Two of the seroconverters (who seroconverted at the 4^th^ ANC visit and 6^th^ PNC visit) vertically transmitted HIV to their babies. Three out of the five women who seroconverted had at least one malaria incident reported during pregnancy. The median age of the women who seroconverted was 20 years but none of them were adolescents (range 18–21). All of them were married and median length of marriage was 1 year (range 0.25–6). None of them had a history of syphilis infection. Seroconversion was detected when a RDT tested positive following previous negative RDTs. Seroconversion occurred at an indeterminate time point between those two testing times, but it was not possible for the research team to determine the exact time of seroconversion. One of the women who seroconverted attended her first ANC during the 1^st^ trimester of her pregnancy (12 weeks) [and seroconverted during her 4^th^ ANC visit]; 3 during the 2^nd^ trimester (16, 20 and 20 weeks) [and seroconverted during the 3^rd^, 2^nd^, and 4^th^ trimesters respectively]; and one during the 3^rd^ trimester (28 weeks) [and had her seroconversion detected at birth].

## Discussion

Our study, which enrolled 1,403 participants (18% adolescent girls and 82% adult women) to investigate the incidence and timing of HIV seroconversion during pregnancy and the postpartum period, found that only five women testing HIV negative during their first ANC visit seroconverted during the ANC and PNC period (incidence proportion 0.41%), with no clear pattern of seroconversion. We also found two cases of vertical HIV transmission among the women who seroconverted. Given the increased risk of HIV transmission during pregnancy in this region, PMTCT efforts should pay close attention to, and discuss as part of the post testing counseling session, the sexual activity of both the women and their partners to identify the appropriate time to repeat HIV testing during ANC and PNC and also promote partner testing among other interventions.

During pregnancy, women do not all stop being sexually active. In our study, 34% of enrolled women reported continuous sexual activity. Only during the last months or weeks of the pregnancy may there be a possibility of interruption. If the spouse or partner has unprotected sexual intercourse with one or more partners during that period and he gets infected with HIV, chances of infection will be high for the pregnant mother and consequently, her baby [11]. This is due to higher viral loads in the weeks following new infections [6–8]. Furthermore, the women may also be engaging in sexual activity with other partners beside their primary partners, as evidenced by the fact that four of the five women who seroconverted in our study reported having an HIV negative primary partner. This finding aligns with a study also conducted in Western Kenya around the same period where none of the women who had an HIV seroconversion during ANC and PNC reported an HIV positive sexual partner [3]. The high rate of unknown partner status may be due to age-discordancy or short duration of partnerships, which is also associated with more external partnerships during ANC and PNC in this population [3].

ANC programs that test women only once during the course of pregnancy may therefore miss an opportunity to uncover the seroconversions taking place after the initial and sometimes only HIV testing. The timing of the additional HIV testing is critical as it has to balance two opposing needs. On the one hand, it has to be carried out soon enough after exposure and infection in order to offer the newly infected mother with all the counseling and treatment available to maintain her health, keep her viral load undetectable and prevent vertical transmission to the baby either in utero or following delivery. On the other hand, programmatically, it has to be offered late enough in the pregnancy in order to diagnose as many women who newly seroconverted as possible. Governments in low-income settings have limited resources for the health of their populations. Ministries of Health (MOHs) often have inadequately low budgets that need to be distributed amongst many health programs. Stock out of commodities, particularly RDTs to test for either HIV or malaria, are very common. Our study aimed to assist MOHs determine whether a second HIV testing should be recommended, and to suggest its timing. In the context of Kenya, where the policy is already in place, and repeat HIV testing after 3 months in the 3^rd^ trimester should be offered to all women whose 1^st^ antenatal test was performed before 28 weeks gestation; our study aimed to determine whether this is the most ideal timing, or if a third test should be provided during the PNC period. In this research project in Kenya, we offered serial HIV testing for pregnant women and new mothers (1,403 adults and 256 adolescents) at 6 time points (1^st^, 2^nd^ 3^rd^ and 4^th^ ANC visits, 6 weeks and 14 weeks postpartum) during ANC and PNC to determine the ideal time to set the additional HIV testing, if any, in case national HIV programs could only institute one additional test. At the outset of the study, five women seroconverted at different time points: one woman tested HIV positive at the 2^nd^ ANC visit, one at the 3^rd^, two at the 4^th^ and one at 6 weeks post-partum. This low number of women who seroconverted does not allow our research team to make a strong generalizable recommendation regarding the best timing for a second HIV test. In our study, the ideal time for a second test was during birth: this would have identified 80% of the seroconversions and allowed to place the mothers and their babies on the PMTCT program.

Our results showed smaller seroconversion rates compared to other studies and regional estimates. A similar study done in the same region in Kenya using serial HIV nucleic acid amplification tests (NAAT) to test for seroconversion during ANC and PNC found three acute infections at enrollment [(those who tested negative by the RDT but positive by the NAAT test), incidence rate 1.21, 95% CI: 0.73–2.01)] and 15 acute infections during follow-up (2 during pregnancy and 13 post-partum) for an incidence rate of 1.23 (95% CI: 0.73–2.02) [3]. The rate of HIV testing for pregnant women during ANC in Kenya was 70% in 2014 (of which 5.6% were found to be HIV positive) and 72% in 2015 (of which 5.3% were found to be HIV positive) [31]. A nationally representative survey conducted in Kenya in 2012 found a 6.1% HIV positivity rate among pregnant women [32]. A meta-analysis using data from 19 cohorts reported a pooled global HIV incidence rate of 3.8/100 person-years [95% confidence interval (95% CI) 3.0–4.6] during pregnancy or postpartum [11]. This means that the use of RDTs could have resulted in us enrolling some women who were already HIV positive but could not be detected by antibody tests or missing women who seroconverted after the 14-week end point.

Among the five women who seroconverted during the study, two women vertically transmitted the virus to their baby, but one of these women declined ART uptake during the period due to denial of her positive status while the other transferred out of the Sauri cluster to enroll at a different facility because of perceived stigma issues. This transfer could have compromised her ART uptake, adherence to ART or support from peers (PMTCT support groups). Our study did prevent transmission to three babies whose mothers would not have otherwise been retested after first ANC.

WHO’s latest guidelines on HIV testing services recommend retesting all HIV-negative pregnant women in the 3rd trimester, postpartum or during labor in the context of a generalized epidemic because of the high risk of acquiring HIV infection during pregnancy [17]. While this would be the appropriate guideline in settings where resources such as diagnostics tests are not limited, it is not realistic in most settings, particularly in sub-Saharan Africa where the HIV epidemic remains the highest. In that context, the additional tests used serially on pregnant women might be more beneficial to other at-risk groups, namely adolescents and men.

Furthermore, our study has shown that it is logistically complicated to systematically test every pregnant woman multiple times: it increases the workload for the nurses, creates an additional challenge with record keeping and can lead to potential provider and client fatigue in re-testing.

In areas where the policy is not specific, and based on local prevalence rates, counties or district health offices need to decide whether to test only once at the first ANC visit, or add a second test. Our results do not suggest a clear pattern of seroconversion during the ANC or PNC period. Another study carried out in the same region of Kenya around the same time as ours also did not find a clear pattern: seroconversions occurred during pregnancy, at 6 weeks, 14 weeks and even 9 months postpartum [3]. Additionally, the low rates of seroconversion we recorded suggest that testing pregnant women multiple times may not be the best use of the commodities. It may be more cost-effective to focus on a sub-population of pregnant women with higher risk than target all women in a low-prevalence setting as was highlighted in a cross-sectional study done among recently delivered mother in Vietnam which showed that women of a lower socio-economic status or with limited opportunities to access PMTCT services were at a higher risk of not being tested for HIV during pregnancy [33]. However, in contexts where incidence is high, a follow up test either during birth or after the mother resumes sexual activity may be protective for the newborn. Repeat testing is recommended in regions with HIV incidence of more than 1 per 100 person-years and has been shown to be cost-effective [17, 34].There is also a need for sustained awareness and education particularly among pregnant women in high prevalence areas on the risk of transmission during the entire pregnancy and PNC period. Finally, in light of the ANC testing rates of 65% at the time of our study (internal MVP data), a strong emphasis also has to be made to ensure all pregnant women get tested at least once in course of their pregnancy.

Finally, we found that three out of the five women who seroconverted over the duration of the study had at least one malaria episode. This is an issue of concern as malaria infection during pregnancy is associated with high risks of both maternal and perinatal morbidity and mortality, and has been associated with miscarriage, premature delivery, low birth weight, congenital infection, and intrauterine demise [35]. Although we did not observe any of these adverse outcomes in our study, it is important for healthcare providers to follow malaria prevention tactics to expecting mothers to avoid complications.

### Strengths and limitations

Our study had several strengths. We followed pregnant women in a rural district a high incidence setting. Prospective cohort design enabled detection of incident HIV throughout pregnancy and up to 6 weeks postpartum. Retention was high at 87%.

Our study also had several limitations. First, we encountered operational challenges due to high staff turnover during our study period. This may have resulted in missing final seroconversion statuses during staff transition periods and therefore incomplete data on key variables that also complicated calculations of incidence rates. Second, while the low number of seroconversions was a great outcome for the women enrolled, it made it difficult for our research team to calculate predictors of seroconversion. That low number could be explained by temporal changes in the country/district regarding ART guidelines which could have heightened awareness in HIV prevention and by the fact that 66% of women enrolled reported no sexual activity during pregnancy, thus they had less opportunities for HIV acquisition. The use of RDTs could have also resulted in us missing acute seroconversions at the end of the PNC period as explained before and it is possible that women who seroconverted were more likely to be the ones who were lost to follow-up. Third, the study experienced lower enrollment of adolescents than planned. Since the research team did not verify the age of participants by requesting identification, it is possible that several adolescents provided a higher age to reduce stigma from early pregnancy. This is especially important, given that about 29% of all HIV infections in Kenya during this time were among adolescents and youth [36] and 15% of women aged 15–19 had already had a birth [37]. It is therefore possible that adolescents may behave differently than found in this study and may be a high-risk group to target for re-testing.

## Conclusion

In the context of low-resource settings, a single HIV test in the course of pregnancy and in the first weeks after birth may not be sufficient. A second test at birth may identify pregnant women who seroconverted after the initial HIV test at first ANC. However, systematic HIV screening at every ANC may unnecessarily use scarce resources that should be redirected to other populations, such as adolescents and men, specifically male partners. Based on the results of our study, and the low number of pregnant women who seroconverted, we believe it would be best to recommend follow-up tests only for high-risk groups.

## Supporting information

S1 File (PDF)

S2 File (PDF)

S1 Data (XLS)

## List of abbreviations

ANC: antenatal care
ART: Antiretroviral therapy
CHW: Community Health Worker
CI: Confidence Interval
HIV: Human Immunodeficiency Virus
MDG: Millennium Development Goals
MOH: Ministry of Health
MTCT: Mother-to-child transmission (of HIV)
MVP: Millennium Villages Project
NAAT: Nucleic Acid Amplification Test
PICT: Provider Initiated Counceling and Testing
PMTCT: Prevention of mother-to-child transmission (of HIV)
PNC: Postnatal Care
RDT: Rapid Diagnostic Test
WHO: World Health Organized
