# Supplementary material for: Optimizing PMTCT efforts by repeat HIV testing during antenatal and perinatal care in resource-limited settings: A longitudinal assessment of HIV seroconversion
Source: PLoS One. 2020 May 29;15(5):e0233396. doi: 10.1371/journal.pone.0233396 (PMC7259594; doi:10.1371/journal.pone.0233396)
Supplement: S1 File — (PDF) [file pone.0233396.s001.pdf]

Patient ID#	D.O.E.[1]	Age	M St[2]	Pr St[3]	Syphilis History[4]	Date of Last MP[5]	1st Visit	2nd Visit		3rd Visit		4th Visit		Birth[6]			6 Weeks			14 Weeks
							GP[7]	GP	St[8]	GP	St	GP	St	GP	St	Sexual Activity[9]	St[10]	FP[11]	Sexual Activity[12]	Status

[1] D.O.E. = Date of Enrollment into study (enter date of 1st ANC visit)
[2] M St = Marital Status (enter **'M'** for married, **'PM'** for polygamous married, **'S'** for single, **'D'** for divorced, **'C'** for cohabiting, **'W'** for widowed, **'WC'** for widowed-cohabiting, **'DC'** for divorced cohabiting)
[3] Pr St = Partner HIV Status (enter verbally reported HIV status of most frequent sexual partner and stage at which status was reported, e.g. for a positive partner status reported at the 1st ANC visit, enter **'+/1',** for a negative partner status reported at birth enter **'–/B'** and for a positive partner status reported 6 weeks after birth enter **'+/6'**)
[4] Enter the patient's confirmed history of syphilis infection (enter **'Y'** for yes and **'N'** for no)
[5] Enter date of last Menstrual Period
[6] Enter the gestation period in weeks at which delivery took place and the mother's HIV status at birth or up to 2 weeks after birth
[7] GP = Gestation Period (enter gestation period in weeks during this visit)
[8] Enter HIV status during this visit (enter **'+'** for positive and **'-'** for negative)
[9] Sexual activity during pregnancy (enter **'Y'** for yes and **'N'** for no)
[10] Enter mother's HIV status 6 weeks after birth (enter **'+'** for positive and **'–'** for negative)
[11] FP = Family Planning administered at 6 weeks after birth and type (enter **'H'** for hormonal, **'NH'** for non-hormonal and **'N/A'** for none administered)
[12] Sexual activity since birth (enter **'Y'** for yes and **'N'** for no)
