# Supplementary material for: Optimizing PMTCT efforts by repeat HIV testing during antenatal and perinatal care in resource-limited settings: A longitudinal assessment of HIV seroconversion
Source: PLoS One. 2020 May 29;15(5):e0233396. doi: 10.1371/journal.pone.0233396 (PMC7259594; doi:10.1371/journal.pone.0233396)
Supplement: S2 File — (PDF) [file pone.0233396.s002.pdf]

Only fill in this manual for all women who test positive for HIV at subsequent ANC visits (besides the first) or at birth or 6 or 14 weeks postpartum, and for the first 20 HIV negative women to complete the study (at 14 weeks post-partum)

Patient ID #	Today's Date	Length of Marriage or Partnership[1]	Condom Use[2]	Malarial Cases During Pregnancy[3]	HIV Status[4]	Partner's Current HIV Status[5]	Eligible Partner on ART[6]

[1] Enter length of current marriage or partnership in years (enter N/A if not married or partnered)

[2] Current condom use (enter **'C'** for consistently, **'O'** for occasionally and **'N'** for never)

[3] Enter number of malarial cases during this pregnancy (BS +ve /Empirically treated)

[4] Enter patient's HIV status during this visit (enter '**+**' for positive and '**-**' for negative)

[5] Enter verbally reported current HIV status of most frequent sexual partner and stage at which status was reported, e.g. for a positive partner status reported at the 2nd ANC visit, enter '**+/2**', for a negative partner status reported at birth enter '**–/B**' and for a positive partner status reported 14 weeks after birth enter **'+/14**'

[6] This column is only to be filled when additional IRB approval is attained about a year from beginning of study. For HIV positive partners, determine whether or not they are on ART care. If eligible (i.e. if partner's CD4 cell count of ≤350 cells/μL) and they are on ART, enter **'Y'** for yes. If partner is ineligible or not on ART, enter **'N'** for no. If partner is HIV negative, enter **'N/A'**.
